# MOC31 for the Diagnosis of Metastatic Carcinoma and Mesothelial Lesions in Effusion Fluid—A Systematic Review and Meta-Analysis

**DOI:** 10.3390/diagnostics15212675

**Published:** 2025-10-23

**Authors:** Alex H. Lin, Matthew Hsu, Joanna K. M. Ng, Sahar J. Farahani, Renald Meçani, Jana Nano, Joshua J. X. Li, Philippe Vielh, Taulant Muka

**Affiliations:** 1 Department of Pathology, School of Clinical Medicine, The University of Hong Kong, Pokfulam, Hong Kongmsshsu@pathology.hku.hk (M.H.);; 2 Department of Pathology and Laboratory Medicine, Memorial Sloan Kettering Cancer Center, New York, NY 10065, USA; jalalis@mskcc.org; 3 Department of Endocrinology and Diabetology, Medical University of Graz, 8010 Graz, Austria; 4 Epistudia, 3008 Bern, Switzerland; 5 Department of Radiation Oncology, TUM School of Medicine and Health, Klinikum Rechts der Isar, Technical University of Munich, 81675 Munich, Germany; 6 Department of Pathology, Medipath and American Hospital of Paris, 92200 Paris, France

**Keywords:** MOC31, immunocytochemistry, serous effusion, metastatic carcinoma, mesothelioma, meta-analysis

## Abstract

**Background/Objectives:** MOC31 immunostain identifies carcinoma cells and is often used in effusion fluid cytology. This systematic review and meta-analysis aim to detail the diagnostic performance of MOC31 with subgroup analysis for different types of carcinomas. **Methods:** A literature search from five databases was performed. Relevant studies were reviewed for the calculation of pooled sensitivity, specificity, positive likelihood ratio (PLR), negative likelihood ratio (NLR), diagnostic odds ratio (DOR), and area under the curve of hierarchical summary receiver operating characteristics (AUC-HSROC). Risk of bias, heterogeneity, and publication bias were assessed by the QUADAS-2, I2 index, and Deeks’ funnel plot. **Results:** In total, 25 studies (10 retrospective cohorts, 10 case–control studies, and 5 case series) were included. The pooled sensitivity, specificity, NLR, PLR, and DOR were 0.926 (0.827–0.971), 0.932 (0.883–0.961), 0.079 (0.005–0.152), 13.610 (5.327–21.892), and 172.475 (83.150–428.100), respectively. The AUC-HSROC was 0.975, indicating excellent performance. Further analysis for adenocarcinomas, mesotheliomas, and benign/reactive mesothelial cells showed sensitivity for adenocarcinomas at 0.962 (0.948–0.975) and specificity for mesotheliomas and mesothelial cells at 0.934 (0.900–0.967) and 0.997 (0.994–1.000). Sensitivity in all four primary site subgroups (female genital, gastrointestinal/hepatobiliary, lung and breast) of adenocarcinoma were high (>0.910). Heterogeneity was observed, and meta-regression identified a trend for the year of publication. No evidence of publication bias was observed. **Conclusions:** Evidence shows that MOC31 could be a robust immunocytochemical marker for identifying and excluding metastatic carcinoma, with excellent diagnostic performance across types of adenocarcinomas. However, evidence is mainly from retrospective studies, highlighting the need for high-quality evidence to further establish MOC31diagnostic utility.

## 1. Introduction

MOC31 is a monoclonal antibody that recognizes epithelial glycoprotein-2, a cell surface antigen associated with carcinoma cells [1]. Cytologic diagnosis of metastatic carcinoma by cytomorphology alone is difficult [2], and immunocytochemistry is often necessary for excluding reactive and/or malignant mesothelial processes [3]. MOC31 sees clinical use in differentiating carcinomas from mesothelial cells and mesotheliomas and is important for the cytologic diagnosis of malignant effusions [4]. The differentiation is clinically important, as the follow-up investigation and management of malignant carcinomatous effusions and mesothelioma are significantly different. Yet, malignant effusions often indicate advanced disease and aggressive tempo, thus, preventing any treatment delay. Prudent and effective use of immunocytochemistry is necessary for accurately selecting the next course of clinical action.

With the availability of alternative carcinoma markers, in particular the more recently available claudin-4 which demonstrated high sensitivity [5], it is important to review the diagnostic performance of the more established MOC31 immunostain as an option for specific clinical scenarios or with claudin-4 or other markers [6]. MOC31 is a strong candidate for such roles due to its robust clinical experience and evidence. This systematic review and meta-analysis aim to detail the diagnostic performance of MOC31 in effusion cytology for malignant carcinomas with subgroup analysis for adenocarcinomas and stratified by primary site. With the multitude of immunostains available for highlighting carcinoma cells [7], each with varying accuracy for different carcinomas, it is important to select the optimal stain or panel of stains catering to each clinical scenario.

## 2. Methods

### 2.1. Study Design

Recent guidelines for conducting systematic reviews and meta-analyses were followed in this study. The Preferred Reporting Items for Systematic Reviews and Meta-Analyses (PRISMA) flowchart and the PRISMA for diagnostic test accuracy (PRISMA-DTA) checklist (Supplementary Material) were used for reporting [8,9].

### 2.2. Literature Search

A literature search of articles up to 29 July 2024 was conducted in bibliographic databases, including Embase, Medline, PubMed, Scopus, and Web of Science. Relevant medical subject headings and search terms were used, such as “MOC31”, “cytology”, “effusion”, “fluid”, “accuracy”, “sensitivity”, “specificity”, and “diagnosis”. The detailed search strategy is provided in the Supplementary Materials. The reference lists of the included studies were also screened for additional studies. EndNote (version 20.1) was used to manage references.

### 2.3. Study Selection

Two independent reviewers completed the screening of titles and abstracts of the identified studies against the predefined inclusion and exclusion criteria. Discrepancies were resolved by discussion or consultation with a third reviewer. Studies were included if (i) they were designed as randomized clinical trials, prospective or retrospective cohort, case–control, or cross-sectional studies, or case series; (ii) had a sample size >= 30; (iii) they assessed the diagnostic accuracy of immunocytochemical marker MOC31 in the diagnosis of carcinoma in effusion fluid cytology; (iv) they reported the frequency of cases and provided histology and/or clinical follow-up data which are regarded as the (combined) reference outcome.

Articles were not eligible for inclusion if (i) they were non-English, review articles, conference abstracts, letters to the editor, editorials, or case reports; (ii) they combined histology or aspiration cytology specimens, and the results of the cytology specimens could not be extracted; (iii) they combined non-carcinomatous malignant effusions such as lymphoma, melanoma, and sarcoma, and the results of the carcinomatous malignant effusions could not be extracted.

### 2.4. Data Extraction and Quality Assessment

Information of the included studies, including study methodology, population demographics, study setting, technical and diagnostic descriptions of cytology preparation and immunocytochemistry procedures, reference standards, and test results (true positive, true negative, false positive, and false negative case counts), as well as measures of frequency, diagnostic, and associations were retrieved by two independent reviewers. All discrepancies were resolved by discussion until a consensus was reached. The QUADAS-2 (Quality Assessment of Diagnostic Accuracy Studies 2) tool was used to assess the risk of bias and applicability of the included studies [10].

### 2.5. Statistical Analysis

The test result data (overall and subgroup case counts for true positive, true negative, false positive, and false negative) of MOC31 expression from the included studies were retrieved and transformed into study-specific two-by-two tables. These tables were used to calculate diagnostic performance metrics, including sensitivity, specificity, positive likelihood ratio (PLR), negative likelihood ratio (NLR), diagnostic odds ratio (DOR), and area under the curve of hierarchical summary receiver operating characteristics (AUC-HSROC). Bivariate random-effects models were applied to estimate both the overall outcome and the subgroup outcomes stratified by study design (retrospective cohort, case–control, and case series). Additionally, univariate fixed- and random-effects models were applied to calculate diagnostic performance metrics for subgroups defined by sample type (adenocarcinoma, benign effusion, and mesothelioma) as well as for subgroups of metastatic carcinomas, classified by the site of the primary tumor as secondary analyses. Statistical heterogeneity was evaluated using Higgins’ I^2^ statistic, with I^2^ values categorized as follows: less than 25% signified low heterogeneity, 25% to 50% signified moderate heterogeneity, and values greater than 50% signified high heterogeneity. The Deeks’ funnel plot and the Deeks’ test statistic were applied to detect publication bias (small study effects). Pre-specified random-effects meta-regression and subgroup analyses were conducted to assess the potential source of heterogeneity (including the study sample size, the year of publication, the study institution, quality of study, and the type of cytologic preparation). All analyses were performed using SPSS (Statistical Product and Service Solutions, version 20) and the following libraries from Python: matplotlib, numpy, scipy, sklearn, statsmodels.

## 3. Results

### 3.1. Study Inclusion

**Figure 1:**
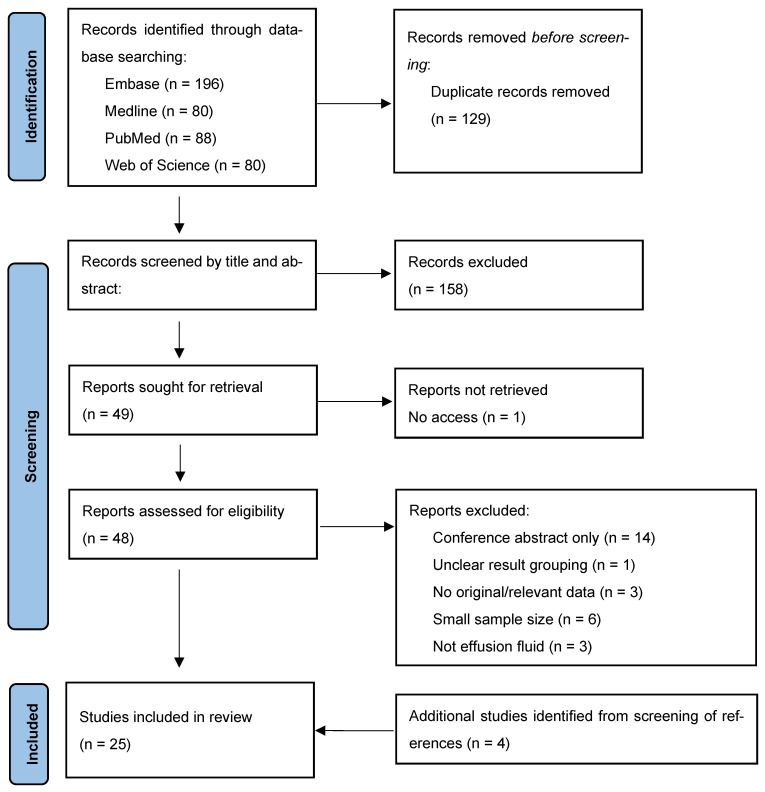
PRISMA flowchart.

A total of 207 studies were identified, and 25 studies (10 retrospective cohorts, 10 case–control studies, and 5 case series) were included in the final analysis [11,12,13,14,15,16,17,18,19,20,21,22,23,24,25,26,27,28,29,30,31,32,33,34,35]. The selection process is presented in the PRISMA flowchart (Figure 1). The studies’ sample sizes varied from 42 to 293, with a total of 3009 cases (specimens), including 1947 metastatic carcinomas and 1062 mesothelioma/benign or reactive effusions (Supplementary Table S1). Most of the studies were conducted in the USA (*n* = 8), followed by China (*n* = 3), the Netherlands (*n* = 3), Greece (*n* = 2), and India (*n* = 2). For quality assessment, the two most common risks of bias in the studies were due to the reference diagnosis not being blinded to assessors (*n* = 12) and non-consecutive case selection (*n* = 10). The QUADAS-2 assessment and study characteristics are detailed in Table 1 and Table 2.

### 3.2. Diagnostic Metrics

**Figure 2:**
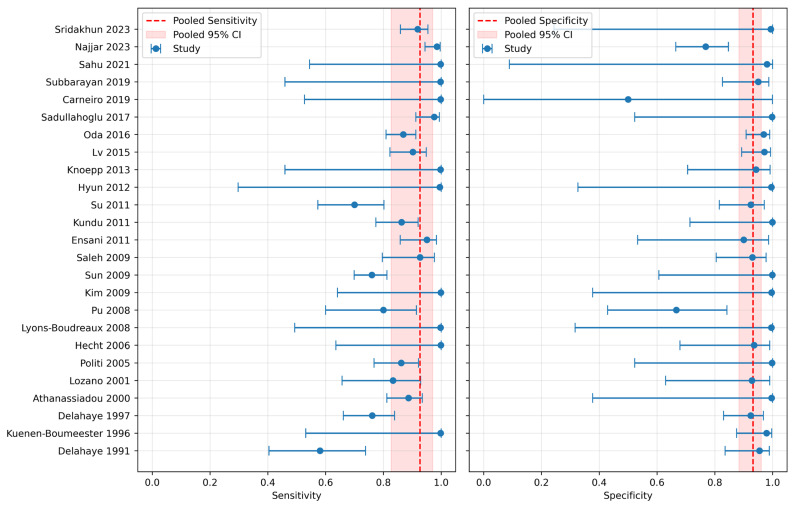
Forest plots of overall sensitivity (0.926, 95% CI: 0.827–0.971) and specificity (0.932, 95% CI: 0.883–0.961) [11,12,13,14,15,16,17,18,19,20,21,22,23,24,25,26,27,28,29,30,31,32,33,34,35].

**Figure 3:**
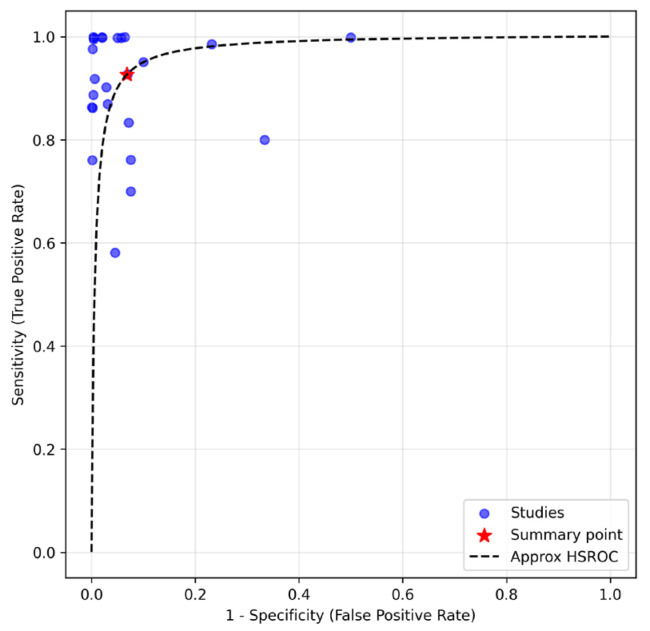
Area under curve of hierarchical summary receiver operating characteristic (AUC-HSROC).

Bivariate random-effects model was used to calculate sensitivity. The overall pooled sensitivities were 0.926 (95% CI: 0.827–0.971). The bivariate random-effects model was also used to calculate specificity, NLR, PLR, and DOR. The overall pooled specificity was 0.932 (95% CI: 0.883–0.961). The NLR, PLR, and DOR were 0.079 (95% CI: 0.005–0.152), 13.610 (95% CI: 5.327–21.892), and 172.475 (95% CI: 83.150–428.100), respectively. The forest plots of sensitivity and specificity are shown in Figure 2. The AUC-HSROC was 0.975, indicating excellent overall diagnostic accuracy for MOC31 immunostain (Figure 3).

Subgroup analysis was performed for studies of the retrospective cohort, case–control and case series. The sensitivity and specificity for the retrospective cohort subgroup in the bivariate random-effects models were 0.909 (95% CI: 0.732–0.973) and 0.954 (95% CI: 0.832–0.989). The sensitivities and specificities for the case–control and case series cohort subgroup in the bivariate random-effects models were 0.950 (95% CI: 0.823–0.987) and 0.934 (95% CI: 0.887–0.962).

Subgroup analysis was performed for the samples of adenocarcinoma, mesothelioma, and benign or reactive mesothelial cells. The sensitivities for the adenocarcinoma subgroup in the fixed- and random-effects models were 0.995 (95% CI: 0.991–0.998) and 0.962 (95% CI: 0.948–0.975), respectively. The specificities for the mesothelioma and benign or reactive mesothelial cells in the random-effects models were 0.912 (95% CI: 0.864–0.960) and 0.996 (95% CI: 0.991–1.000).

There were 1265 cases of metastatic adenocarcinoma with retrievable primary site and staining result, including from the most frequent to the least—lung (*n* = 480), gastrointestinal/hepatobiliary tract (*n* = 264), female genital organs (*n* = 252), breast (*n* = 190), genitourinary (*n* = 11), thyroid (*n* = 2), and head and neck (*n* = 1). The highest sensitivity was seen in metastatic adenocarcinomas of the primary female genital (0.983, 95% CI: 0.964–1.000), followed by gastrointestinal/hepatobiliary tract (0.958, 95% CI: 0.927–0.988), lung (0.944, 95% CI: 0.915–0.973), and breast (0.917, 95% CI: 0.864–0.970) (Supplementary Figure S1).

High and moderate statistical heterogeneity was observed in pooled estimates (pooled sensitivity: I^2^ = 74.9%, τ^2^ = 4.831, 95% prediction interval: 0.106–0.999; pooled specificity: I^2^ = 54.4%, τ^2^ = 1.385, 95%, prediction interval: 0.526–0.994). To explore potential sources of this heterogeneity, a meta-regression analysis was conducted. Among the variables examined (year of publication, study sample size, study institution, overall study quality (risk of bias and applicability concerns in QUADAS-2 assessment), and the type of cytologic preparation), only the year of publication showed a trend towards significance (*p* = 0.094).

### 3.3. Publication Bias

**Figure 4:**
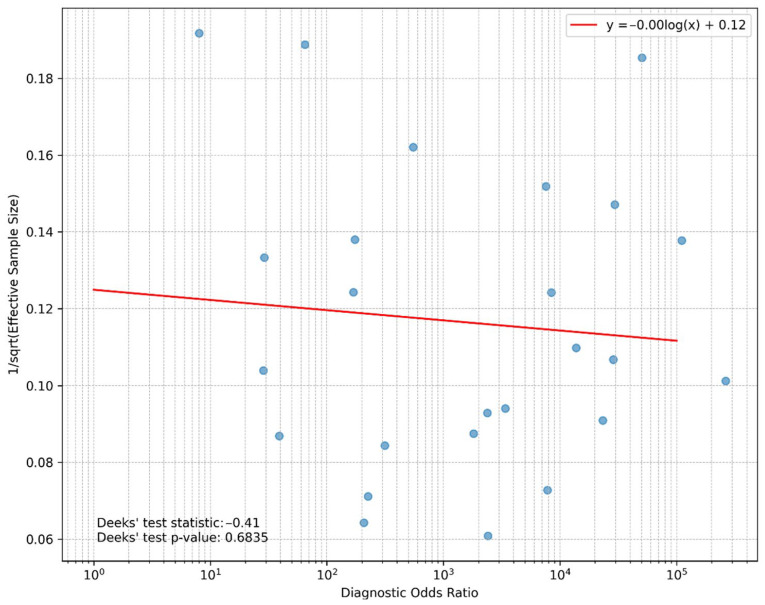
Deeks’ funnel plot.

The publication bias of the included studies was assessed using Deeks’ test. The Deeks’ funnel plot did not show significant asymmetry by visual inspection, and the Deeks’ test *p*-value (*p* = 0.684) indicated a low risk of publication bias (Figure 4).

## 4. Discussion

Malignant effusions can be associated with a multitude of causes, with the most frequent being metastatic carcinomas [36], while melanomas, sarcomas, and lymphomas are significantly less encountered in malignant effusions [4,37]. The accurate diagnosis of malignant carcinomatous effusion is a clinical priority. Cytological specimens can be harvested for a multitude of theragnostic tests [38,39], and with the availability of effective targeted therapy for late stage malignancies [40], the survival of patients with malignant effusions has improved significantly [41].

Carcinomas from the lung and breast show the highest propensity in metastasizing to serous fluid cavities [4,36], which is indirectly reflected by the distribution of cases with a specified primary site in the current study, with lung and breast ranking first and fourth in subgroup case number. Similar to the current findings, gastrointestinal and female genital carcinomas are also commonly seen in malignant carcinomatous effusion [36]. The diagnostic performance of immunocytochemical stains is affected by disease factors such as tumor type, cellularity, and sample composition [5].

Findings from the current study demonstrated that MOC31 immunostain exhibits excellent diagnostic accuracy for identifying metastatic carcinoma in effusion fluid cytology, with a pooled sensitivity, specificity, and AUC-HSROC all exceeding 0.950 (Figure 2). The PLR and DOR were also correspondingly high and the NLR low, supporting its overall robustness. Importantly, MOC31 maintained high sensitivity across all adenocarcinoma subgroups, with values above 0.910, and even reached 0.990 in metastatic disease from female genital primaries—a statistically significant increase compared to breast and lung primaries. This is complemented by an excellent specificity to mesothelioma of 0.912, supporting its clinical utility in differentiating metastatic carcinomas, including lung adenocarcinomas, from mesotheliomas. A recent meta-analysis performed by Kleinaki et al. reported a sensitivity of claudin-4 at 0.980 [42]. As such, if a laboratory has a tested and established protocol for MOC31, it may not be recommendable to simply replace MOC31 with claudin-4, but to complement the detection of metastatic carcinoma by both markers.

However, some carcinoma types may exhibit reduced immunoreactivity to MOC31. For example, neuroendocrine neoplasms display less intense staining to epithelial markers [43]. These potential pitfalls can be avoided by recognizing the limitations of MOC31 in certain tumors. However, the literature on the performance of MOC31 on uncommon carcinomas is scarce, and the current systematic review was unable to address the issue. It may be prudent to include more disease-specific immunostains under clinical contexts with sufficient degree of suspicion and to supplement MOC31 with mesothelial markers to reduce the risk of an erroneous false negative interpretation.

The presence of high statistical heterogeneity across pooled estimates warrants careful interpretation of the results. A meta-regression was performed to explore potential sources of this variability. Only the year of publication showed a borderline association with heterogeneity, suggesting that evolving diagnostic practices or immunostaining protocols over time may have influenced study outcomes. Other factors did not significantly explain the observed variation. Beyond these, unmeasured differences in immunocytochemical techniques, such as antibody clone, dilution, staining intensity thresholds, and interpretation criteria, likely contributed further to inter-study variability. These methodological inconsistencies, coupled with geographic and institutional differences, highlight the need for standardized approaches to immunostaining and result interpretation to enhance reproducibility and facilitate more reliable comparisons across future diagnostic accuracy studies.

### Limitations

Nearly half of the included studies (48%) lacked blinding between the index test and reference standard, introducing a risk of confirmation bias that may have led to overestimation of diagnostic accuracy. Additionally, 40% of the studies employed non-consecutive or selective case inclusion, increasing the likelihood of spectrum bias by favoring diagnostically clear cases. These methodological shortcomings limit the generalizability of the findings and highlight the need for more rigorously designed studies with blinded assessments and unbiased patient selection.

One notable limitation in the current meta-analysis is the variability in the definition and interpretation of MOC31 positivity across studies, which introduces a potential threshold effect. While some studies considered any level of membranous or cytoplasmic staining as positive, others applied stricter criteria, requiring moderate or strong intensity in more than 10% of cells. These discrepancies can lead to misclassification and potentially affect pooled diagnostic estimates, particularly sensitivity. In addition, not all studies reported thresholds and limited comparative cutoff appraisal. Importantly, differences in threshold criteria may also account for part of the observed heterogeneity across studies.

## 5. Conclusions

MOC31 immunostain is a robust immunocytochemical marker for identifying metastatic carcinoma in effusion fluid cytology, with sensitivity, specificity, and AUC-HSROC all exceeding 0.950. The excellent diagnostic performance is maintained in metastatic adenocarcinomas and all subgroups stratified by primary site (female genital organs, gastrointestinal/hepatobiliary tract, lung and breast). Unless other malignant neoplasms such as lymphomas, sarcomas or melanomas, or rare carcinomas are suspected, MOC31 should be adequate for both diagnosing and excluding most of the common metastatic carcinomas. Future studies should adopt uniform definitions of positivity or perform comparative validation of cutoff values to facilitate more accurate pooled analyses.

## Figures and Tables

**Table 1:** Characteristics and risk of bias assessment of the studies included.

				Risk of Bias				Applicability Concerns		
	Size	Year	Location	Patient Selection	Index Test	Reference Standard	Flow and Timing	Patient Selection	Index Test	Reference Standard
Sridakhun 2023 [11]	152	2023	Thailand	L	L	H	L	L	L	L
Najjar 2023 [12]	220	2023	USA	L	L	H	L	L	L	L
Sahu 2021 [13]	64	2021	India	L	U	H	L	L	L	L
Subbarayan 2019 [14]	84	2023	India	L	L	H	L	L	L	L
Carneiro 2019 [15]	55	2019	Brazil	U	L	L	U	L	L	L
Sadullahoglu 2017 [16]	142	2017	Turkey	L	L	H	L	L	H	L
Oda 2016 [17]	265	2016	Japan	L	L	L	L	L	L	L
Lv 2015 [18]	162	2015	China	L	L	H	L	L	L	L
Knoepp 2013 [19]	61	2013	USA	H	H	H	H	L	H	L
Hyun 2012 [20]	45	2012	USA	H	L	L	L	L	L	L
Su 2011 [21]	71	2011	China	L	L	L	L	L	L	L
Kundu 2011 [22]	113	2011	USA	U	L	L	U	L	L	L
Ensani 2011 [23]	211	2011	Iran	L	L	L	L	L	L	L
Saleh 2009 [24]	84	2009	USA	L	L	L	L	L	L	L
Sun 2009 [25]	118	2009	China	L	L	L	L	L	L	L
Kim 2009 [26]	293	2009	Korea	L	L	H	L	L	L	L
Pu 2008 [27]	43	2008	USA	H	L	L	U	L	L	L
Lyons-Boudreaux 2008 [28]	71	2008	USA	L	L	U	L	L	L	L
Hecht 2006 [29]	103	2006	USA	H	L	L	U	L	L	L
Politi 2005 [30]	134	2005	Greece	H	L	L	U	L	L	L
Lozano 2001 [31]	44	2001	Spain	H	L	H	U	L	L	L
Athanassiadou 2000 [32]	137	2000	Greece	H	L	H	U	L	L	L
Delahaye 1997 [33]	154	1997	The Netherlands	L	L	L	U	L	L	L
Kuenen-Boumeester 1996 [34]	108	1996	The Netherlands	H	L	L	U	L	L	L
Delahaye 1991 [35]	75	1991	The Netherlands	H	L	H	U	L	H	L

H—high risk, L—low risk, U—unclear risk.

**Table 2:** Technical specifications of immunocytochemistry of the studies included.

	Preparation	Source	Dilution	Intensity	Percentage	Pattern
Sridakhun 2023 [11]	Cell block	Novocastra	1:25	NS *	NS	Membranous
Najjar 2023 [12]	Cell block	Dako	1:50	NS	NS	Membranous and/or cytoplasmic
Sahu 2021 [13]	Cell block	NS	NS	At least weak	NS	Membranous and/or cytoplasmic
Subbarayan 2019 [14]	Smear	Bio SB	NS	NS	>20%	Membranous and cytoplasmic
Carneiro 2019 [15]	Cell block	Dako	1:200	At least weak	>0%	Membranous
Sadullahoglu 2017 [16]	Smear	Eurodiagnostica	1:40	At least moderate	NS	NS
Oda 2016 [17]	Cell block	Dako	1:50	At least weak	>0%	Membranous
Lv 2015 [18]	Smear	Eurodiagnostica	1:10	NS	NS	Membranous and cytoplasmic
Knoepp 2013 [19]	Smear	Dako	1:100	NS	NS	NS
Hyun 2012 [20]	Cell block	Dako	1:160	NS	>10%	Membranous and/or cytoplasmic
Su 2011 [21]	Smear	Eurodiagnostica	1:20	NS	NS	Membranous
Kundu 2011 [22]	Cell block	Maixin Bio	Prediluted	At least weak	>10%	Membranous and/or cytoplasmic
Ensani 2011 [23]	Cell block	Novocastra	1:50	NS	NS	Membranous
Saleh 2009 [24]	Cell block	Dako	NS	NS	NS	Membranous
Sun 2009 [25]	Cell block	Maixin Bio	Prediluted	At least weak	>10%	Membranous and/or cytoplasmic
Kim 2009 [26]	Cell block	Dako	1:100	NS	>5%	Membrane and cytoplasmic
Pu 2008 [27]	Cell block	Dako	1:30	NS	>10%	Membranous or cytoplasmic
Lyons-Boudreaux 2008 [28]	Cell block	Dako	1:10	At least weak	NS	Membranous
Hecht 2006 [29]	Cell block	Biogenic	Prediluted	NS	>5%	Membranous, cytoplasmic or nuclear
Politi 2005 [30]	Cell block	Dako	1:60	At least weak	NS	Membranous
Lozano 2001 [31]	Cytospin	Dako	1:200	NS	NS	Membranous and/or cytoplasmic
Athanassiadou 2000 [32]	Smear	Biogenex	Prediluted	At least weak	>10%	Membranous, cytoplasmic, or nuclear
Delahaye 1997 [33]	Cell block	Dako	1:200	Any intensity	>0%	Membranous
Kuenen-Boumeester 1996 [34]	Cell block	Dako	1:100	NS	NS	Membranous and/or cytoplasmic
Delahaye 1991 [35]	Smear	Organon	1:10	NS	NS	NS

* NS—not specified.

## Data Availability

The original contributions presented in this study are included in the article/Supplementary Material. Further inquiries can be directed to the corresponding author.

## Supplementary Materials

The following supporting information can be downloaded at https://www.mdpi.com/article/10.3390/diagnostics15212675/s1, Supplementary Materials S1: PRISMA-DTA Checklist, Supplementary Materials S2: Search algorithms, Supplementary Table S1: two-by-two tables, supplementary Figure S1: Forest plots.

## References

[1] Zimmermann S., Wels W., Froesch B.A., Gerstmayer B., Stahel R.A., Zangemeister-Wittke U. (1997). A novel immunotoxin recognising the epithelial glycoprotein-2 has potent antitumoural activity on chemotherapy-resistant lung cancer. Cancer Immunol. Immunother..

[2] Pairman L., Beckert L.E.L., Dagger M., Maze M.J. (2022). Evaluation of pleural fluid cytology for the diagnosis of malignant pleural effusion: A retrospective cohort study. Intern. Med. J..

[3] Shital P., Mirza M., Gondhali G. (2017). Pleural fluid ‘cell block’ analysis in malignant pleural effusion: Sensitive, superior over fluid cytology and suitable for immunohistochemistry analysis (IHC), will decrease need for thoracoscopy guided procedures. Eur. Respir. J..

[4] Poon I.K., Chan R.C.K., Choi J.S.H., Ng J.K.M., Tang K.T., Wong Y.Y.H., Chan K.P., Yip W.H., Tse G.M., Li J.J.X. (2023). A comparative study of diagnostic accuracy in 3026 pleural biopsies and matched pleural effusion cytology with clinical correlation. Cancer Med..

[5] Li J.J.X., Ng J.K.M., Tsang J.Y., Tsang Y.T., Mak K.F., Tse G.M. (2024). Comparison of Claudin-4, BerEP4, Carcinoembryonic Antigen and MOC31 in Serous Fluids Metastases Demonstrate High Sensitivity of Claudin-4 at Low Cellularity. Diagn. Cytopathol..

[6] Li J.J.X., Cheng H.Y., Lee C.H.C., Ng J.K.M., Tsang J.Y., Tse G.M. (2025). Single-cell multiplex immunocytochemistry in cell block preparations of metastatic breast cancer confirms sensitivity of GATA-binding protein 3 over gross cystic disease fluid protein 15 and mammaglobin. Cancer Cytopathol..

[7] Lin A.H., Hsu M., Ng J.K.M., Farahani S.J., Li J.J.X., Nano J., Raeisi-Dehkordi H., Tang W., Vielh P., Muka T. (2025). Performance of the Monoclonal Antibody B72.3 in Diagnosis of Malignant Carcinomatous Serous Effusions-A Systematic Review and Meta-Analysis of Diagnostic Performance. Cytopathology.

[8] Page M.J., McKenzie J.E., Bossuyt P.M., Boutron I., Hoffmann T.C., Mulrow C.D., Shamseer L., Tetzlaff J.M., Akl E.A., Brennan S.E. (2021). The PRISMA 2020 statement: An updated guideline for reporting systematic reviews. BMJ.

[9] McInnes M.D.F., Moher D., Thombs B.D., McGrath T.A., Bossuyt P.M., Clifford T., Cohen J.F., Deeks J.J., Gatsonis C., Hooft L. (2018). Preferred Reporting Items for a Systematic Review and Meta-analysis of Diagnostic Test Accuracy Studies: The PRISMA-DTA Statement. JAMA.

[10] Whiting P.F., Rutjes A.W., Westwood M.E., Mallett S., Deeks J.J., Reitsma J.B., Leeflang M.M., Sterne J.A., Bossuyt P.M. (2011). QUADAS-2: A revised tool for the quality assessment of diagnostic accuracy studies. Ann. Intern. Med..

[11] Sridakhun N., Intarawichian P., Thanee M., Watcharadetwittaya S. (2023). Diagnosis of Metastatic Carcinoma Using Body Cavity Fluid Specimens: A Comparison of Diagnostic Panels. Acta Cytol..

[12] Najjar S., Gan Q., Stewart J., Sneige N. (2023). The utility of claudin-4 versus MOC-31 and Ber-EP4 in the diagnosis of metastatic carcinoma in cytology specimens. Cancer Cytopathol..

[13] Sahu S., Sharma S., Gupta P., Dey P. (2021). MOC31 Immunostaining in the Diagnosis of Metastatic Adenocarcinoma in Serous Fluid: Special Emphasis on Atypical Cytological Cases. Acta Cytol..

[14] Subbarayan D., Bhattacharya J., Rani P., Khuraijam B., Jain S. (2019). Use of Panel of Markers in Serous Effusion to Distinguish Reactive Mesothelial Cells from Adenocarcinoma. J. Cytol..

[15] Carneiro F.P., Muniz-Junqueira M.I., De Vasconcelos Carneiro M., De Araújo Oliveira Í., Soares A.C., De Vargas Haar N., Takano G.H.S., De Sousa Vianna L.M., De Carvalho Caldas G., Vieira D.L.M. (2019). Anti-EpCAM antibodies for detection of metastatic carcinoma in effusions and peritoneal wash. Oncol. Lett..

[16] Sadullahoglu C., Nart D., Veral A. (2017). The importance of EZH2 and MOC-31 expression in the differential diagnosis of benign and malignant effusions. Diagn. Cytopathol..

[17] Oda T., Ogata S., Kawaguchi S., Minabe S., Dokyu M., Takahashi H., Kumazawa F., Shimazaki H., Takano M., Hase K. (2016). Immunocytochemical utility of claudin-4 versus those of Ber-EP4 and MOC-31 in effusion cytology. Diagn. Cytopathol..

[18] Lv M., Leng J.H., Hao Y.Y., Sun Y., Cha N., Wu G.P. (2015). Expression and significance of MOC-31 and calretinin in pleural fluid of patients with lung cancer. Diagn. Cytopathol..

[19] Knoepp S.M., Placido J., Fields K.L., Thomas D., Roh M.H. (2013). The application of immunocytochemistry to direct smears in the diagnosis of effusions. Diagn. Cytopathol..

[20] Hyun T.S., Barnes M., Tabatabai Z.L. (2012). The diagnostic utility of D2-40, calretinin, CK5/6, desmin and MOC-31 in the differentiation of mesothelioma from adenocarcinoma in pleural effusion cytology. Acta Cytol..

[21] Su X.Y., Li G.D., Liu W.P., Xie B., Jiang Y.H. (2011). Cytological differential diagnosis among adenocarcinoma, epithelial mesothelioma, and reactive mesothelial cells in serous effusions by immunocytochemistry. Diagn. Cytopathol..

[22] Kundu U.R., Krishnamurthy S. (2011). Use of the monoclonal antibody MOC-31 as an immunomarker for detecting metastatic adenocarcinoma in effusion cytology. Cancer Cytopathol..

[23] Ensani F., Nematizadeh F., Irvanlou G. (2011). Accuracy of immunohistochemistry in evaluation of malignant pleural and peritoneal effusions. Pol. J. Pathol..

[24] Saleh H.A., El-Fakharany M., Makki H., Kadhim A., Masood S. (2009). Differentiating reactive mesothelial cells from metastatic adenocarcinoma in serous effusions: The utility of immunocytochemical panel in the differential diagnosis. Diagn. Cytopathol..

[25] Sun Y., Wu G.P., Fang C.Q., Liu S.L. (2009). Diagnostic utility of MOC-31, HBME-1 and MOC-31 mRNA in distinguishing between carcinoma cells and reactive mesothelial cells in pleural effusions. Acta Cytol..

[26] Kim J.H., Kim G.E., Choi Y.D., Lee J.S., Lee J.H., Nam J.H., Choi C. (2009). Immunocytochemical panel for distinguishing between adenocarcinomas and reactive mesothelial cells in effusion cell blocks. Diagn. Cytopathol..

[27] Pu R.T., Pang Y., Michael C.W. (2008). Utility of WT-1, p63, MOC31, mesothelin, and cytokeratin (K903 and CK5/6) immunostains in differentiating adenocarcinoma, squamous cell carcinoma, and malignant mesothelioma in effusions. Diagn. Cytopathol..

[28] Lyons-Boudreaux V., Mody D.R., Zhai J., Coffey D. (2008). Cytologic malignancy versus benignancy: How useful are the “newer” markers in body fluid cytology?. Arch. Pathol. Lab. Med..

[29] Hecht J.L., Pinkus J.L., Pinkus G.S. (2006). Monoclonal antibody MOC-31 reactivity as a marker for adenocarcinoma in cytologic preparations. Cancer.

[30] Politi E., Kandaraki C., Apostolopoulou C., Kyritsi T., Koutselini H. (2005). Immunocytochemical panel for distinguishing between carcinoma and reactive mesothelial cells in body cavity fluids. Diagn. Cytopathol..

[31] Lozano M.D., Panizo A., Toledo G.R., Sola J.J., Pardo-Mindán J. (2001). Immunocytochemistry in the differential diagnosis of serous effusions: A comparative evaluation of eight monoclonal antibodies in Papanicolaou stained smears. Cancer.

[32] Athanassiadou P., Gonidi M., Liossi A., Petrakakou E., Nakopoulou L., Zerva C., Athanassiades P. (2000). Moc-31, fibronectin and CEA in the differential diagnosis of malignant effusions: An immunocytochemical study. Pathol. Oncol. Res..

[33] Delahaye M., van der Ham F., van der Kwast T.H. (1997). Complementary value of five carcinoma markers for the diagnosis of malignant mesothelioma, adenocarcinoma metastasis, and reactive mesothelium in serous effusions. Diagn. Cytopathol..

[34] Kuenen-Boumeester V., van Loenen P., de Bruijn E.M., Henzen-Logmans S.C. (1996). Quality control of immunocytochemical staining of effusions using a standardized method of cell processing. Acta Cytol..

[35] Delahaye M., Hoogsteden H.C., Van der Kwast T.H. (1991). Immunocytochemistry of malignant mesothelioma: OV632 as a marker of malignant mesothelioma. J. Pathol..

[36] Jovanovic D. (2020). Etiopathogenesis of malignant pleural effusion. AME Med. J..

[37] Li J.J.X., Chan W.C., Chau H.H.L., Wu C., Tse G.M. (2019). Cytologic diagnosis of metastatic malignant phyllodes tumor of the breast in pleural effusion. Diagn. Cytopathol..

[38] Ng J.K.M., Chow C., Chan R.C.K., Chan K.P., Li J.J.X., Li M.S.C., To K.-F. (2022). EGFR testing in paraffin-embedded cell block cytology material is reliable with increased detection for effusion fluid. Lung Cancer.

[39] Wei S., Talarchek J.N., Huang M., Gong Y., Du F., Ehya H., Flieder D.B., Patchefsky A.S., Wasik M.A., Pei J. (2023). Cell block-based RNA next generation sequencing for detection of gene fusions in lung adenocarcinoma: An institutional experience. Cytopathology.

[40] Tang F.H., Wong H.Y.T., Tsang P.S.W., Yau M., Tam S.Y., Law L., Yau K., Wong J., Farah F.H.M., Wong J. (2025). Recent advancements in lung cancer research: A narrative review. Transl. Lung Cancer Res..

[41] Chow L.H., Ngai A.M.Y., Tang C.Y., Lee J.H.S., Lee A.L.H., Li J.J.X., Ip P.P.C. (2025). Malignant ascites in ovarian cancer is compatible with long-term (10 year) survival with associations to clinicopathological features. J. Ovarian Res..

[42] Kleinaki M., Vey J.A., Awounvo S., Ishak A., Arnaouti M., Ryu H.S., Nikas I.P. (2025). The Diagnostic Accuracy of Claudin-4 Immunochemistry in Differentiating Metastatic Carcinomas From Mesothelial Processes in Serous Effusion Cytology: A Systematic Review and Meta-analysis. Arch. Pathol. Lab. Med..

[43] Lam P.P.H., Lum R.T.W., Chan J.W.Y., Lau R.W.H., Ng C.S.H., Li J.J.X. (2025). Neuroendocrine Lesions Arising From Mediastinal Teratoma—A Case Report and Literature Review. Int. J. Surg. Pathol..

