# Supplementary material for: MOC31 for the Diagnosis of Metastatic Carcinoma and Mesothelial Lesions in Effusion Fluid—A Systematic Review and Meta-Analysis
Source: Diagnostics (Basel). 2025 Oct 23;15(21):2675. doi: 10.3390/diagnostics15212675 (PMC12608982; doi:10.3390/diagnostics15212675)

1. Adenocarcinoma (sensitivity)

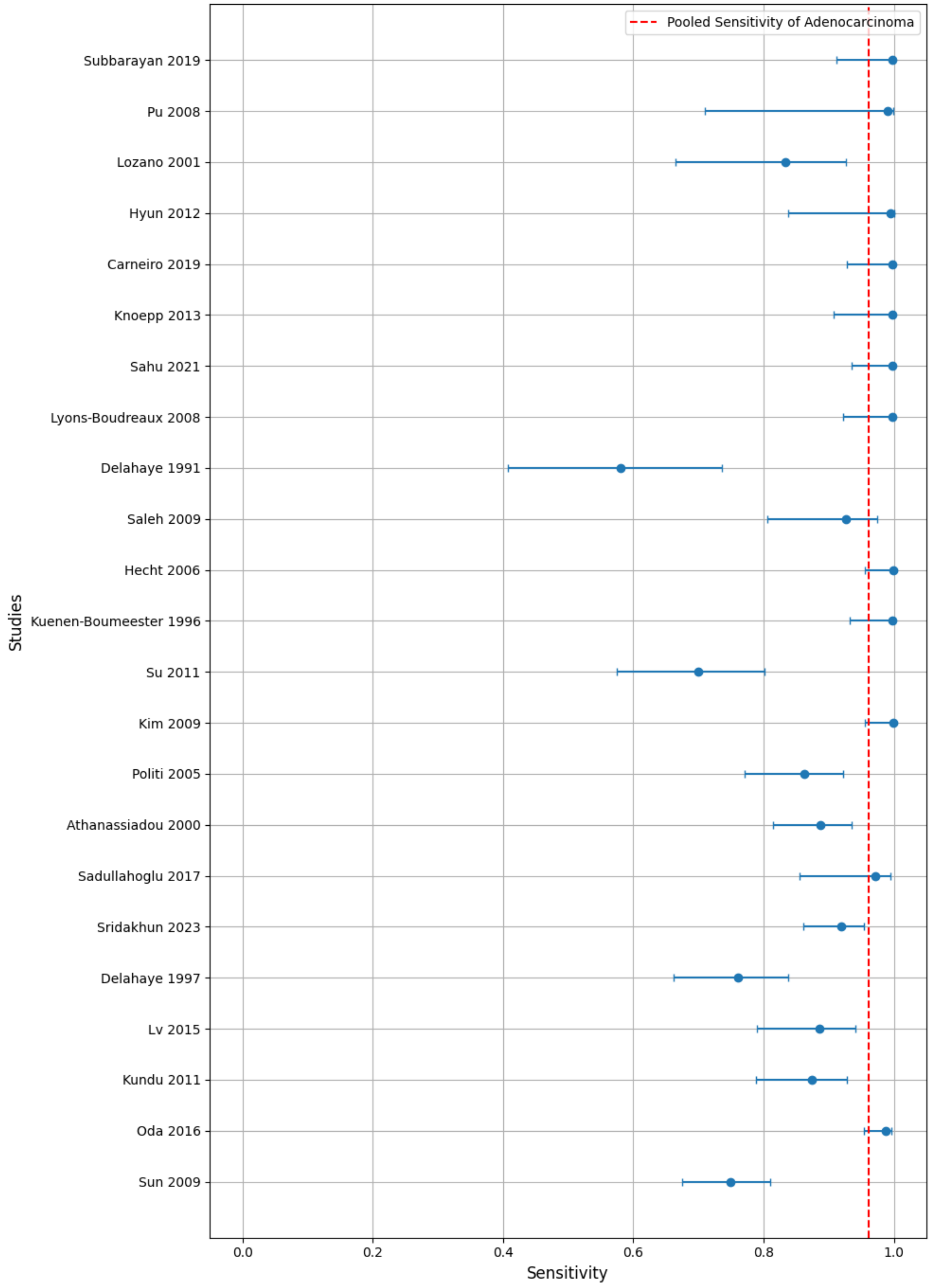

* Random effects / fixed effects model

2. Benign effusion (specificity)

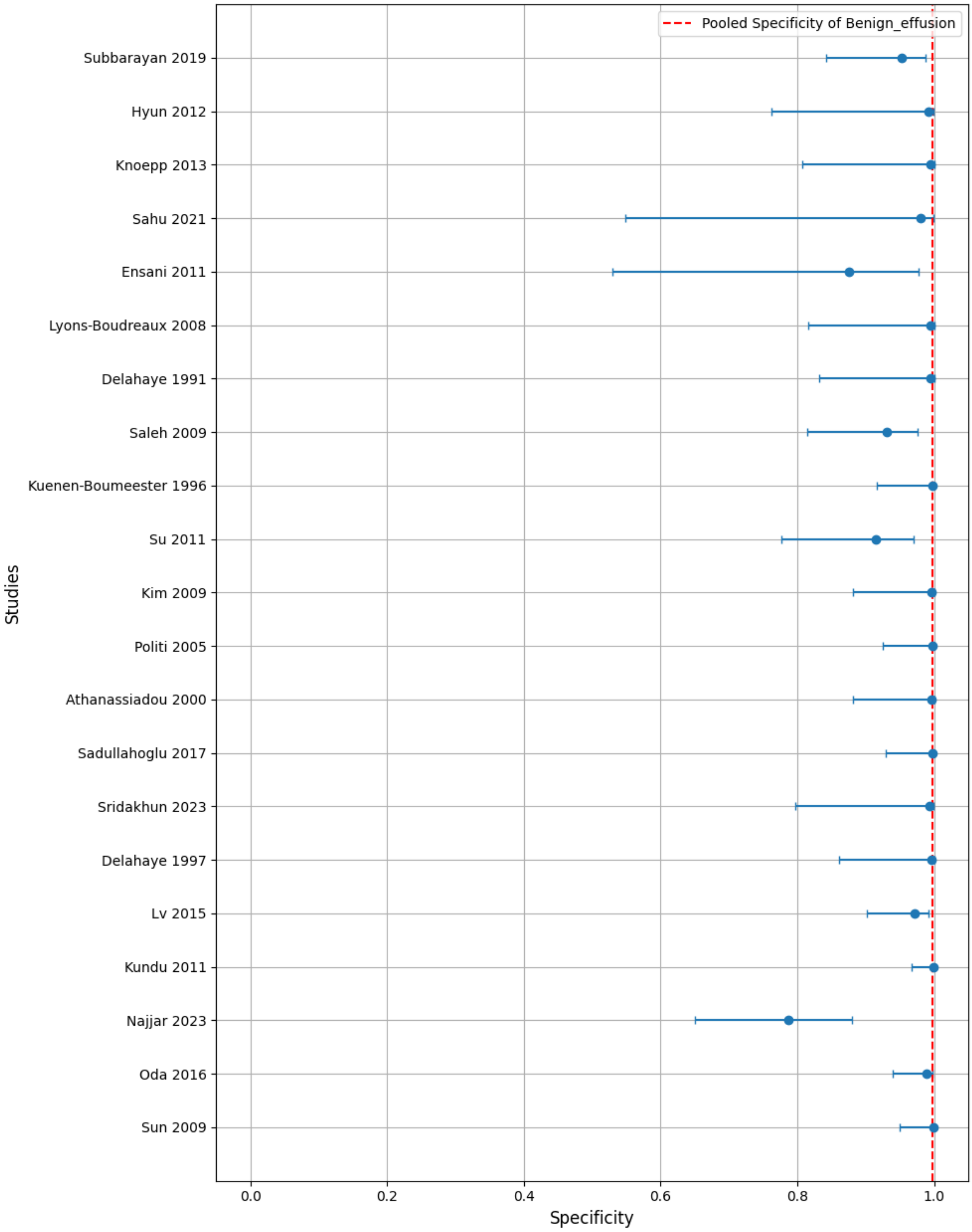

3. Mesothelioma (specificity)

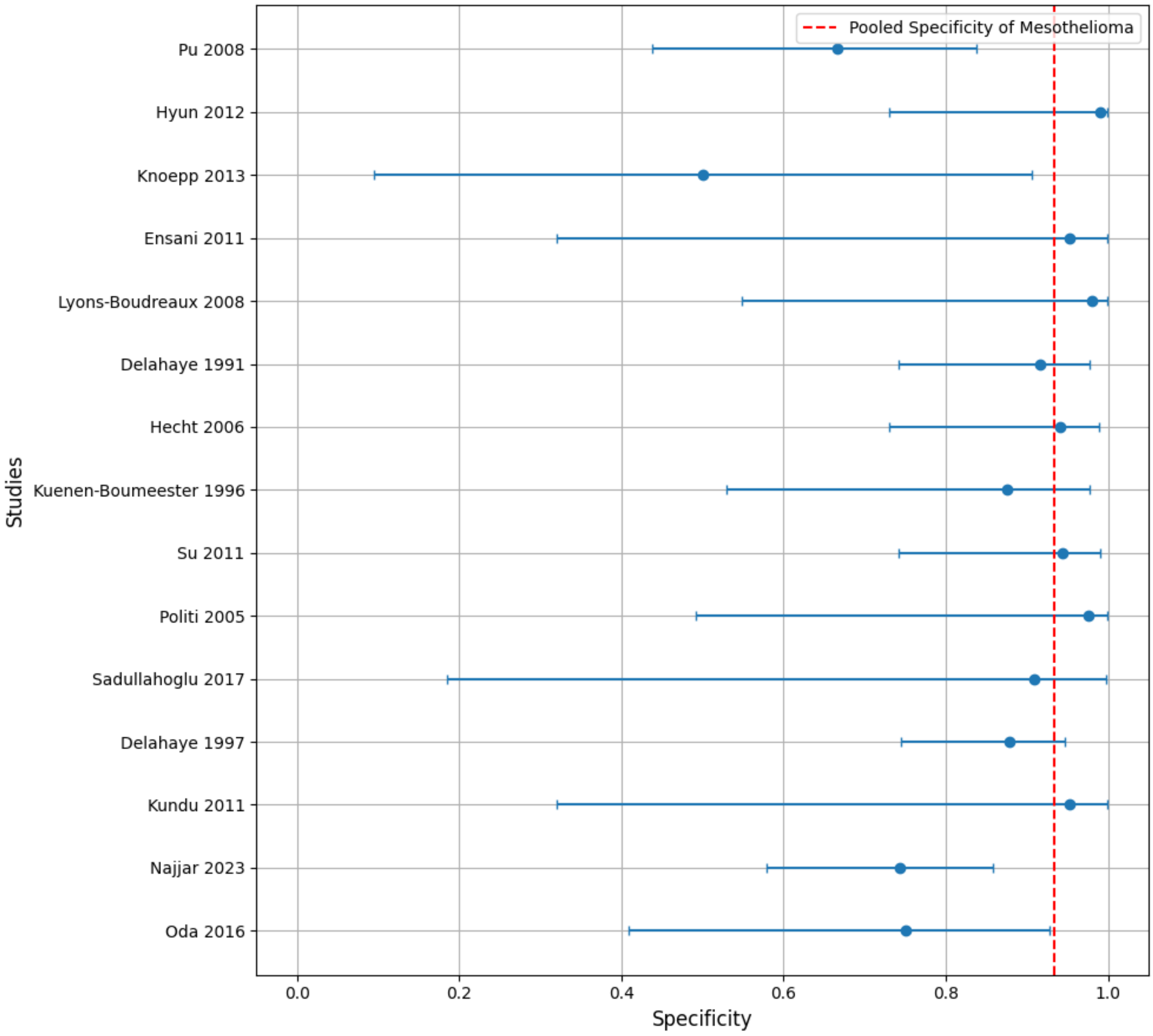

4. Lung adenocarcinoma (sensitivity)

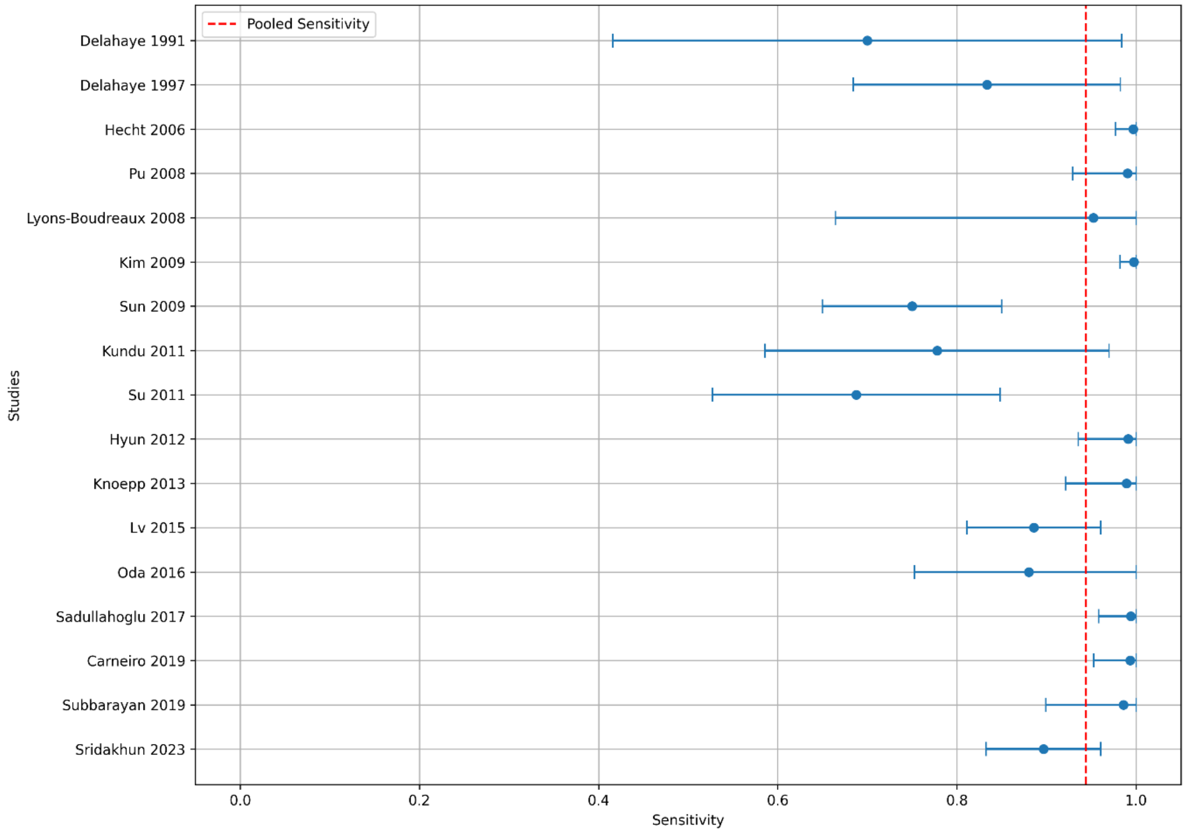

5. Gastrointestinal / hepatobiliary adenocarcinoma (sensitivity)

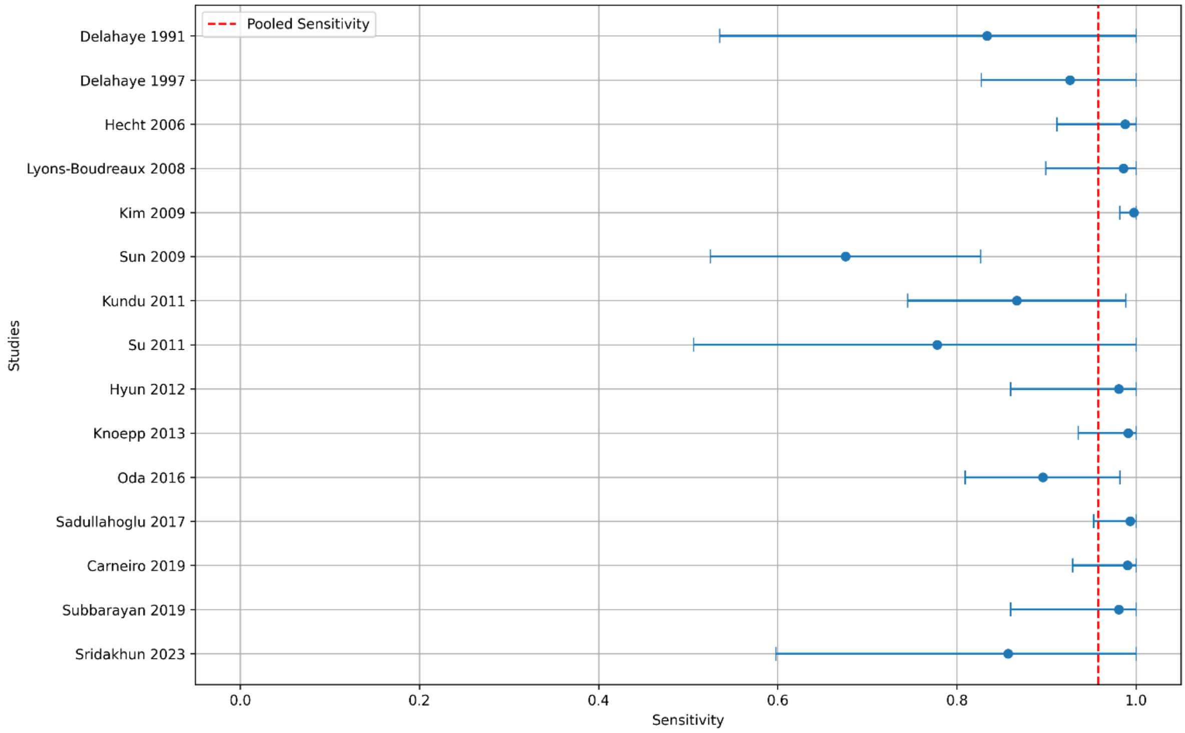

6. Female genital adenocarcinoma (sensitivity)

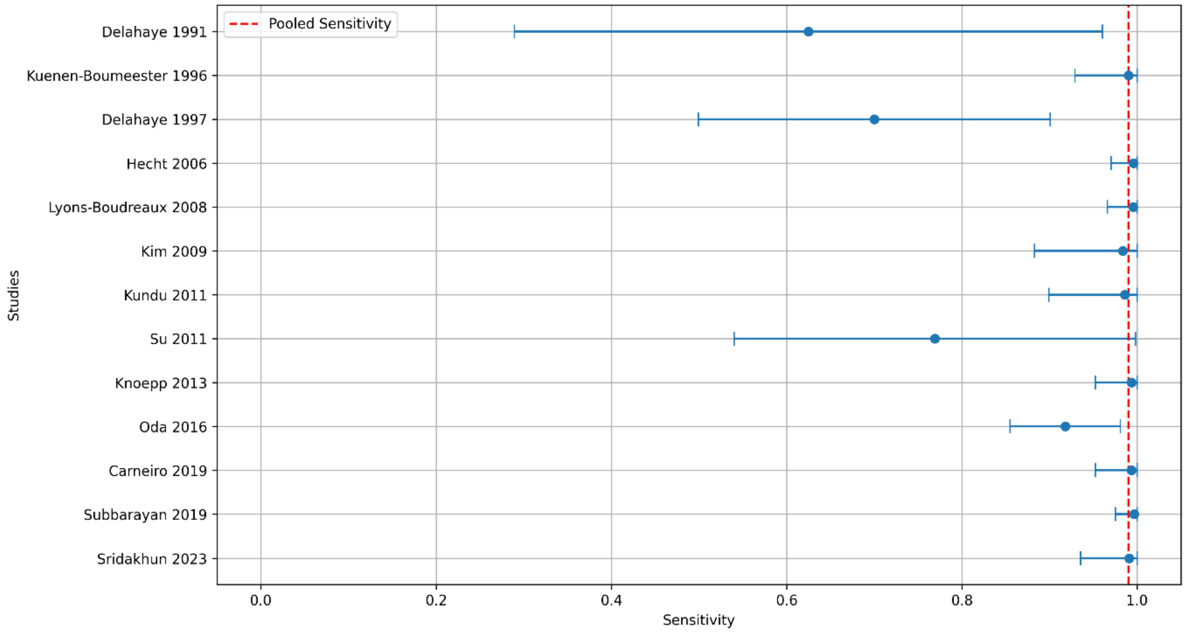

7. Breast adenocarcinoma (sensitivity)

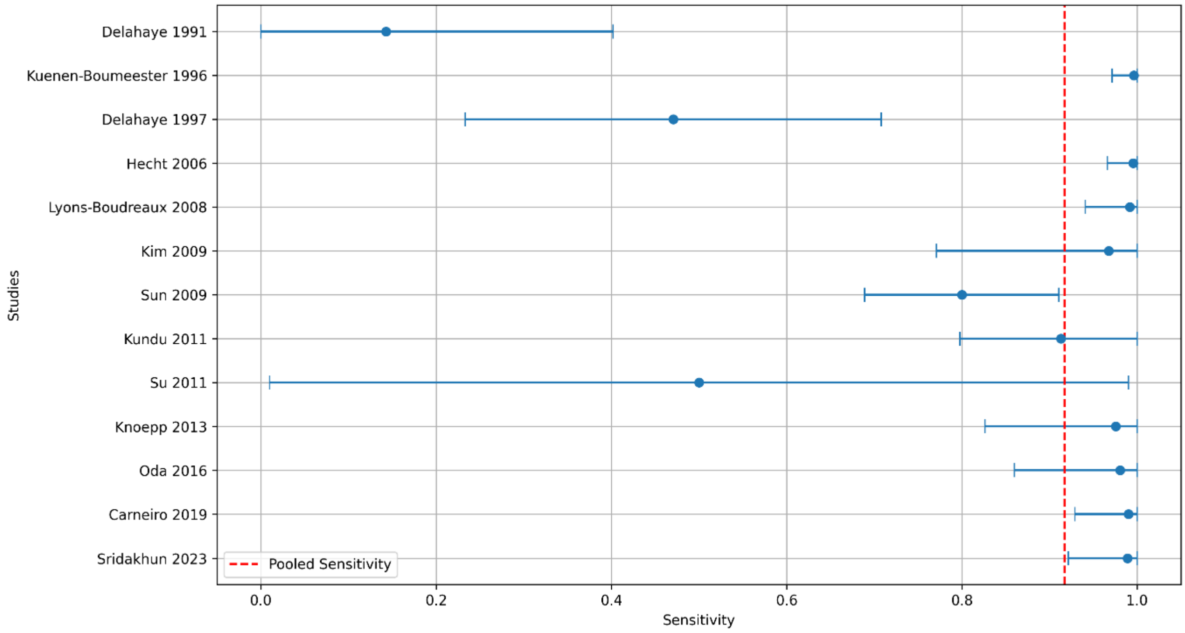

Supplement: Supplementary file 1 [file diagnostics-15-02675-s001.zip › #Supplementary_figure_S1.pdf]
