# Supplementary material for: MOC31 for the Diagnosis of Metastatic Carcinoma and Mesothelial Lesions in Effusion Fluid—A Systematic Review and Meta-Analysis
Source: Diagnostics (Basel). 2025 Oct 23;15(21):2675. doi: 10.3390/diagnostics15212675 (PMC12608982; doi:10.3390/diagnostics15212675)
Supplement: Supplementary file 1 [file diagnostics-15-02675-s001.zip › #Supplementary_material_S2.pdf]

Supplementary material S2. Search algorithms

PubMed

("MOC-31"[tiab] OR "MOC31"[tiab]) AND (“cytology”[Mesh] OR “cytolog*”[tiab] OR “pleural effusion”[Mesh] OR “pericardial effusion”[Mesh] OR “ascitic fluid”[Mesh] OR “effusion”[tiab] OR “fluid”[tiab]) AND ("sensitivity and specificity"[Mesh] OR "sensiti*"[tiab] OR "specifici*"[tiab] OR “accura*”[tiab] OR “odds ratio”[Mesh] OR “odds*”[tiab] OR “risk”[Mesh] OR “risk*” [tiab] OR “predict*”[tiab] OR “predictive value of tests”[Mesh] OR “analys*”[tiab] OR “ratio*”[tiab] OR “PPV”[tiab] OR “NPV”[tiab] OR "diagnosis"[Mesh] OR "diagno*"[tiab])

WOS

("MOC-31" OR "MOC31") AND (“cytology” OR “cytolog*” OR “pleural effusion” OR “ascitic fluid” OR “effusion” OR “fluid”) AND ("sensitivity and specificity" OR "sensiti*" OR "specifici*" OR “accura*” OR “odds*” OR “risk*” OR “predict*” OR “predictive value of tests” OR “analys*” OR “ratio*” OR “PPV” OR “NPV” OR "diagnosis" OR "diagno*")

Medline

(MOC31 or MOC-31).mp. AND (Cytology/ or cytolog*.mp. or Pleural Effusion/ or Pericardial Effusion/ or Ascitic Fluid/ or effusion.mp. or fluid.mp.) AND ("Sensitivity and Specificity"/ or sensiti*.mp. or specifici*.mp. or accura*.mp. or Odds Ratio/ or odds*.mp. or Risk/ or risk*.mp. or predict*.mp. or “Predictive Value of Tests”/ or analys*.mp. or ratio*.mp. or PPV.mp. or NPV.mp. or Diagnosis/ or diagno*.mp.)

Scopus

(MOC-31 OR MOC31) AND (cytology OR cytolog* OR pleural effusion OR ascitic fluid OR effusion OR fluid) AND (sensitivity and specificity OR sensiti* OR specifici* OR accura* OR odds* OR risk* OR predict* OR analys* OR ratio* OR PPV OR NPV OR diagnosis OR diagno*)

Embase

(MOC31 or MOC-31).mp. AND (Cytology/ or cytolog*.mp. or Pleural Effusion/ or Pericardial Effusion/ or Ascitic Fluid/ or effusion.mp. or fluid.mp.) AND ("Sensitivity and Specificity"/ or sensiti*.mp. or specifici*.mp. or accura*.mp. or Odds Ratio/ or odds*.mp. or Risk/ or risk*.mp. or predict*.mp. or “Predictive Value of Tests”/ or analys*.mp. or ratio*.mp. or PPV.mp. or NPV.mp. or Diagnosis/ or diagno*.mp.)
