# Supplementary material for: MOC31 for the Diagnosis of Metastatic Carcinoma and Mesothelial Lesions in Effusion Fluid—A Systematic Review and Meta-Analysis
Source: Diagnostics (Basel). 2025 Oct 23;15(21):2675. doi: 10.3390/diagnostics15212675 (PMC12608982; doi:10.3390/diagnostics15212675)
Supplement: Supplementary file 1 [file diagnostics-15-02675-s001.zip › Supplementary_table_S1_2x2tables.pdf]

Supplementary material Table S1. 2 x 2 table

	Year	True Positive	False Negative	True Negative	False Positive
Sridakhun 2023 (11)	2023	125	9	16	0
Najjar 2023 (12)	2023	NA	NA	63	19
Sahu 2021 (13)	2021	59	0	5	0
Subbarayan 2019 (14)	2023	42	0	NA	NA
Carneiro 2019 (15)	2019	55	0	NA	NA
Sadullahoglu 2017 (16)	2017	86	2	54	0
Oda 2016 (17)	2016	163	6	97	0
Lv 2015 (18)	2015	53	39	68	2
Knoepp 2013 (19)	2013	43	1	17	0
Hyun 2012 (20)	2012	21	0	24	0
Su 2011 (21)	2011	42	18	49	4
Kundu 2011 (22)	2011	76	12	123	0
Ensani 2011 (23)	2011	58	3	9	1
Saleh 2009 (24)	2009	38	3	40	3
Sun 2009 (25)	2009	76	0	165	52
Kim 2009 (26)	2009	88	0	30	0
Pu 2008 (27)	2008	20	5	12	6
Lyons-Boudreaux 2008 (28)	2008	48	0	24	0
Hecht 2006 (29)	2006	86	0	16	1
Politi 2005 (30)	2005	69	11	54	0
Lozano 2001 (31)	2001	25	5	13	1
Athanassiadou 2000 (32)	2000	95	12	30	0
Delahaye 1997 (33)	1997	67	21	61	5
Kuenen-Boumeester 1996 (34)	1996	56	0	51	1
Delahaye 1991 (35)	1991	18	13	42	2

*Studies with NA were single arm studies consisting of mesothelial or carcinomatous effusions only
